# Role of Probiotics in Gut Microbiome and Metabolome in Non-Alcoholic Fatty Liver Disease Mouse Model: A Comparative Study

**DOI:** 10.3390/microorganisms12051020

**Published:** 2024-05-17

**Authors:** Tian Wu, Zheng Zeng, Yanyan Yu

**Affiliations:** Department of Infectious Diseases, Peking University First Hospital, Beijing 100034, China; wutian2020@bjmu.edu.cn

**Keywords:** NAFLD, *Akkermansia muciniphila*, VSL#3, gut microbiota, metabolomics

## Abstract

Non-alcoholic fatty liver disease (NAFLD) is the most prevalent chronic liver condition worldwide. Numerous studies conducted recently have demonstrated a connection between the dysbiosis of the development of NAFLD and gut microbiota. Rebuilding a healthy gut ecology has been proposed as a strategy involving the use of probiotics. The purpose of this work is to investigate and compare the function of probiotics *Akkermansia muciniphila* (*A. muciniphila*) and VSL#3 in NAFLD mice. Rodent NAFLD was modeled using a methionine choline-deficient diet (MCD) with/without oral probiotic delivery. Subsequently, qPCR, histological staining, and liver function tests were conducted. Mass spectrometry-based analysis and 16S rDNA gene sequencing were used to investigate the liver metabolome and gut microbiota. We found that while both *A. muciniphila* and VSL#3 reduced hepatic fat content, *A. muciniphila* outperformed VSL#3. Furthermore, probiotic treatment restored the β diversity of the gut flora and *A. muciniphila* decreased the abundance of pathogenic bacteria such as *Ileibacterium valens*. These probiotics altered the metabolism in MCD mice, especially the glycerophospholipid metabolism. In conclusion, our findings distinguished the role of *A. muciniphila* and VSL#3 in NAFLD and indicated that oral-gavage probiotics remodel gut microbiota and improve metabolism, raising the possibility of using probiotics in the cure of NAFLD.

## 1. Introduction

Non-alcoholic fatty liver disease (NAFLD) is becoming an epidemic all over the world, with a prevalence ranging from 14% to more than 30% [1,2,3]. NAFLD is a general term for several different liver conditions, including non-alcoholic steatohepatitis (NASH). NASH can lead to liver cirrhosis and potentially hepatocellular carcinoma (HCC) [4]. The process by which NAFLD develops is intricate, and its exact mechanism is unknown. The “multi-hit” approach is currently well-accepted, and research has pointed out that the gut flora is essential in the pathophysiology of NAFLD [5,6]. Since the intestinal flora and its metabolites influence the pathophysiological processes of the liver through the gut–liver axis, gut dysbiosis can contribute to type 2 diabetes (T2DM), metabolic syndrome, obesity, and NAFLD [7,8]. In turn, patients with NAFLD have shown variations in their abundance and taxa of gut microbiota [9]. Extensive research has been conducted on the possibility of preventing and treating NAFLD by modulating the gut microbiota, including the application of probiotics [10,11,12] or prebiotics [13], and fecal microbiome transplantation [14].

Probiotics are live bacteria that provide health benefits to their hosts [15]. Recently, probiotics have been employed to treat a variety of diseases such as acute gastroenteritis, antibiotic-associated diarrhea [16], and some metabolic diseases. For example, *Lactobacillus rhamnosus* GG (LGG), a classic probiotic strain, has been reported to reduce liver fat accumulation and reverse fructose-induced NAFLD in mice by upregulating liver fibroblast growth factor 21 expression [17]. Crystal et al. found that dietary intake of *Lactococcus lactis Subspecies cremoris* could reduce weight gain and liver steatosis, lower serum cholesterol levels, and increase glucose tolerance in mice fed with a Western diet [12]. In a double-blind randomized controlled clinical trial, insulin sensitivity was improved in some participants with T2DM after 12 weeks of *Lactobacillus reuteri* DSM 17938 intake [18]. Furthermore, it was reported that supplementation of *Bifidobacterium longum OLP-01*, combined with exercise, in diabetes animal models could reduce fasting blood glucose, alleviate liver damage, and repair pancreatic damage [19]. Additionally, considering the characteristics of the anti-oxidation stress of probiotics and its role in cardiovascular disease-related risk factors such as diabetes and obesity, probiotics have the potential to be applied in cardiovascular disease [20,21].

In addition to the extensively studied classic probiotics mentioned above, *Akkermansia muciniphila* (*A. muciniphila*) has attracted attention in recent years. *A. muciniphila* belongs to the Verrucomicrobia phylum and was first discovered in human feces in 2004 [22]. *A. muciniphila* supplementation improves glucose homeostasis and increases thermogenesis in diet-induced obese mice by inducing the secretion of glucose-like peptide-1-inducing protein [23]. In addition, *A. muciniphila* has been reported to alleviate liver steatosis and inflammation, improve bile acid metabolism, and alleviate intestinal cell apoptosis induced by oxidative stress by regulating L-aspartate metabolism in obese mice given a high-fat and high-cholesterol diet [24]. It was also reported that oral administration of *A. muciniphila* could significantly reduce serum triglycerides (TG), prevent fatty liver, improve metabolism, and restore gut microbiota diversity in mice [25,26,27]. Therefore, *A. muciniphila* is regarded as a promising next-generation probiotic due to its ability to prevent and treat T2DM, as well as other metabolic disorders [28].

Three strains of Bifidobacterium (*B. longum*, *B. breve*, and *B. infantis*), one strain of Streptococcus thermophilus BT01, and four strains of *Lactobacillus* (*L. casei*, *L. plantarum*, *L. acidophilus*, and *L. delbrueckii* subsp. *bulgaricus*) make up VSL#3, a combination of high-dose probiotics. Colitis can be effectively treated with VSL#3 [29]. Clinical trial outcomes for NAFLD patients, however, are not always consistent. In obese children with lower body mass index (BMI), a 4-month administration of VSL#3 dramatically reduced the severity of NAFLD and decreased BMI, while another trial in Latino teenagers found that treatment with VSL#3 resulted in significant increases in trunk and total adiposity [10,30]. On the other hand, VSL#3 may play a beneficial role in animal experiments, including ameliorating MCD diet-induced liver fibrosis, and reducing hepatic fat content in Farnesoid X receptor (FXR) knockout mice that are given a Western diet [31,32].

Both *A. muciniphila* and VSL#3 have been reported to affect metabolic disorders; however, there is no research comparing the effectiveness of the two probiotics in NAFLD. Using a mouse model, this study examines and compares the effects of both probiotics on NASH as well as the impacts of *Akkermansia muciniphila* and VSL#3 on intestinal flora and metabolism.

## 2. Materials and Methods

### 2.1. Animal Models

Six-week-old male C57BL/6 J mice were procured from ViewSolid Biotech Co., Ltd., located in Beijing, China. All mice were kept in ventilated cages with specified pathogen-free (SPF) conditions, 12 h light/dark cycles, enriched water, and unlimited food. A standard diet (SD) or a methionine- and choline-deficient (MCD) diet was given to the mice. Following a week of acclimatization to the study’s setting, mice were randomly divided into four groups: SD group, MCD group, MCD+*A. muciniphila* group (MCD_A), and MCD+VSL#3 group (MCD_V). Xietong Biotech Co., Ltd. (Nanjing, China) supplied all the diets. The SD group and MCD group received 200 μL normal saline treatment daily, while the MCD_A group received 200 μL normal saline gavage-fed *A. muciniphila* (2 × 10^8^ CFU, purchased from the American Type Culture Collection, BAA-835) and the MCD_V group received 200 μL normal saline gavage-fed VSL#3 (2.25 × 10^9^ CFU, purchased from De Simone, Seoul, Republic of Korea) for 8 weeks. Weekly body weight measurements were made. For microbial analysis, the animal fecal pellets were gathered and quickly frozen in liquid nitrogen. Following a heart puncture to obtain blood samples, these 40 mice were killed, and their livers were gathered and weighed. Tissues from the intestine were aseptically removed and kept cold until needed. The Peking University First Hospital Ethics Committee acknowledged all animal protocols (No. J2022142).

### 2.2. Analyses Biochemical

The Department of Laboratory at Servicebio Technology Co., Ltd. (Wuhan, China) measured serum levels of alanine aminotransferase (ALT), triglyceride (TG), low-density lipoprotein (LDL), and high-density lipoprotein (HDL) with an automatic biochemical analyzer (Chemray 800, Rayto, Shenzhen, China), and using corresponding commercial kits.

### 2.3. Histological Examination

Samples of liver and intestinal tissue were embedded in paraffin, preserved in 4% paraformaldehyde, cut into 4–5 μm slices, and stained with hematoxylin and eosin (HE) for histological examination (using a kit from Beyotime, Beijing, China). By staining the liver using Oil Red O Staining, lipid buildup in the liver was examined (kit bought from Beyotime, Beijing, China).

### 2.4. RNA Extraction and RT–qPCR Analysis

RNAisoPlus (TaKaRa, Tokyo, Japan) was used to extract RNA from frozen liver tissues in accordance with the operating manual. With the use of a PrimeScriptTM RT reagent Kit from Takara, reverse transcription PCR was conducted. The cDNA was used for q-PCR with TB Green^®^ Premix Ex TaqTM (TaKaRa) with an Applied Biosystems 7500 Real-Time PCR System (Applied Biosystems, Hercules, CA, USA). Our data was standardized using the expression of glyceraldehyde-3-phosphate dehydrogenase (GAPDH). Tianyi Huiyuan Biotech Co., Ltd. (Beijing, China) supplied the primers. In Table 1, the primer sequences are displayed.

### 2.5. Gut Microbial Sequencing and Data Processing

Using the cetyltrimethylammonium bromide (CTAB) technique, mice feces were used for the extraction of genomic DNA. On a 1% agarose gel, the concentration and purity of DNA were observed. Using sterile water, DNA was diluted to 1 ng/ffL based on the concentration. The gene of 16S rDNA was amplified with primers (515F–806R). Wuhan, China-based Metware Biotechnology Co., Ltd. provided the sequencing service. Metware Cloud online (https://cloud.metware.cn/, accessed on 30 November 2023), an online bioinformatic tool, was used to perform further analyses including diversity analysis, main coordinates analysis, relative abundance analysis, and others. The raw data of the sequencing were uploaded to the Sequence Read Archive (SRA) database of NCBI (PRJNA1097335).

### 2.6. Hepatic Metabolomics Analysis

Extraction of metabolites was performed from the liver samples (∼20 mg). The TripleTOF 6600+ mass spectrometer (SCIEX, Foster City, CA, USA), coupled with an LC-30A system (Shimadzu, Kyoto, Japan), was used for the untargeted metabolomics profiling by liquid chromatography–tandem mass spectrometry (LC-MS/MS) system. On a Waters ACQUITY Premier HSS T3 Column 1.8 µm (2.1 mm * 100 mm ) at 40 °C, chromatographic separation was accomplished. At a flow rate of 0.4 mL/min, the mobile phase was composed of 0.1% formic acid/water (A) and 0.1% formic acid/acetonitrile (B). The database of the Metware Biotechnology Co., Ltd. (Wuhan, China) and multiple reaction monitoring (MRM) were the bases for the qualitative and quantitative study of metabolites.

### 2.7. Statistical Analysis

The means ± SD are used to express all data. One-way analysis of variance (ANOVA) and Tukey’s post hoc test were used for the statistical analysis. With the aid of GraphPad Prism 9 and R4.2.0, the data were plotted graphically. At *p* < 0.05, differences were deemed statistically significant.

## 3. Results

### 3.1. A. muciniphila Has a Stronger Effect on Reducing Liver Lipid Accumulation in NAFLD Mice Than Does VSL#3

**Figure 1:**
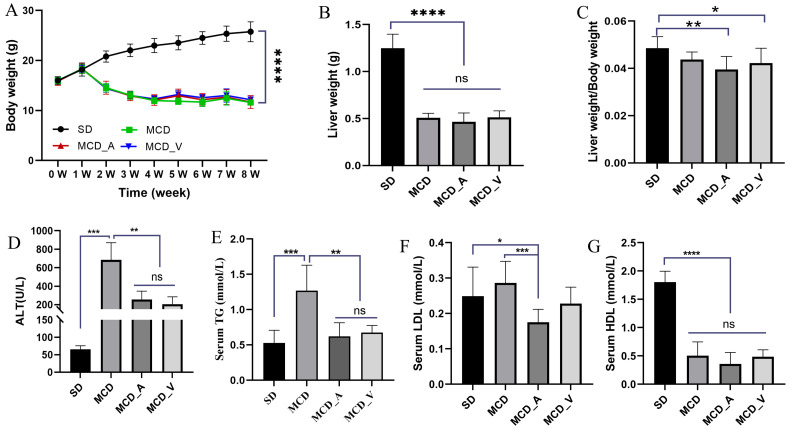
Probiotics improve liver functions and reduce lipid accumulation. (**A**) Alterations in body weight over 8 weeks; (**B**) Liver weight of mice in four groups; (**C**) The ratio of liver weight/body weight; (**D**) Liver function ALT for mice in each group; (**E**) Serum TG levels, (**F**) serum LDL levels, and (**G**) serum HDL levels for mice in each group. The above values are expressed as mean ± SD, *n* = 10; ns, no significant difference; * *p* < 0.05; ** *p* < 0.01; *** *p* < 0.001; **** *p* < 0.0001. ALT, alanine aminotransferase; TG, triglyceride.

**Figure 2:**
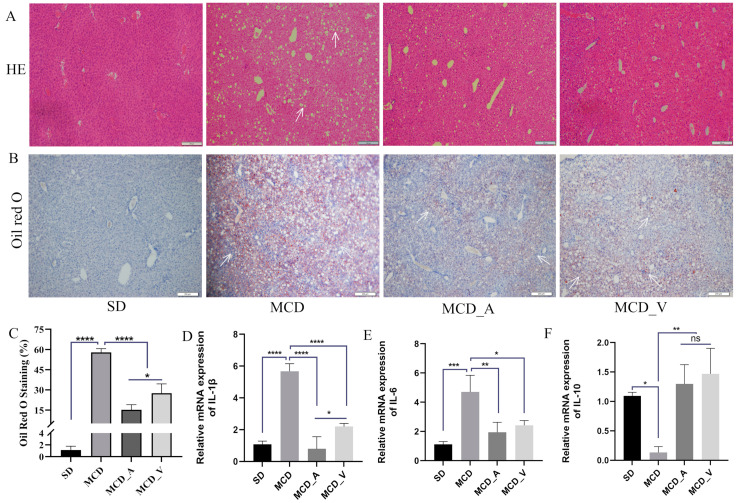
*A. muciniphila* and VSL#3 reduce lipid deposition and inflammation in the liver. (**A**,**B**) Hepatic histological presentation by Oil Red O and HE staining. White arrows indicate hepatocyte ballooning and hepatocyte steatosis. (**C**) Quantitative analysis of Oil Red O staining. (**D**–**F**) The expression of IL-1b, IL-6, and IL-10 in livers of mice. The above values are expressed as mean ± SD, *n* = 10; ns, no significant difference; * *p* < 0.05; ** *p* < 0.01; *** *p* < 0.001; **** *p* < 0.0001.

Mice were given either SD or MCD for eight weeks, at which point the mice were collected to investigate the influence of *A. muciniphila* or VSL#3 supplements in the progress of fibrosis and steatosis in NASH. Every week, the body weight was recorded. As seen in Figure 1A, mice fed MCD had a consistent decrease in body weight. In addition, the average weight of livers in SD group mice was markedly higher than the other three groups, and the ratio of liver/body weight was higher compared with MCD_A and MCD_V groups. Additionally, Figure 1B,C show that the application of *A. muciniphila* or VSL#3 did not affect body weight, liver weight, or their ratio. Additionally, as indicated in Figure 1D,G, measurements of the serum TG and ALT were made. MCD dramatically increased ALT levels compared to SD and oral probiotics groups (Figure 1D). Similarly, the application of *A. muciniphila* or VSL#3 decreased the blood lipids in mice, as revealed by a significantly lower level of TG identified in MCD_A and MCD_V groups, as shown in Figure 1E. There are no differences among SD, MCD_A, and MCD_V as to ALT and TG levels. Figure 1F,G show the levels of lipoproteins LDL and HDL. The content of LDL in the MCD_A group was decreased significantly compared with the SD and MCD groups; however, the levels of HDL in mice fed MCD were lower than in the SD group, indicating that probiotic application did not affect HDL. As indicated in Figure 2A, mice given MCD showed increased hepatocyte ballooning and hepatocyte steatosis, as demonstrated by histological analysis of liver sections, suggesting that MCD generated NASH models. Probiotics, however, lessened these pathogenic alterations in the MCD_A and MCD_V groups. Lipid droplets more clearly formed in mice fed with MCD after 8 weeks, compared to mice in SD group, as demonstrated by oil red O staining. Supplements with *A. muciniphila* or VSL#3 dramatically reduced liver steatosis, as seen in Figure 2B. Moreover, *A. muciniphila* outperformed VSL#3 as to lowering the amount of liver fat (Figure 2C). Furthermore, qPCR analysis revealed that mice fed MCD had much higher expression of IL-1b than other three groups (Figure 2D); in contrast, IL-1b dramatically decreased in the MCD_A and MCD_V when compared with MCD group, and its expression was lower in MCD_A group. As demonstrated in Figure 2E, the liver IL-6 levels were similar to those for IL-1β, however, there is no difference between the MCD_A and MCD_V groups. The expression levels of TNF-α and IL8 are shown in Supplementary Figure S1A,B. Although their expression increases in the MCD group, the difference is not statistically significant. Both probiotics greatly decreased hepatic inflammation, yet the expression of IL-10 was entirely different from those of IL-1b and IL-6 (Figure 2F).

### 3.2. VSL#3 Treatment Improved Intestinal Damage and Gut Barrier Function Better, as Compared to A. muciniphila

**Figure 3:**
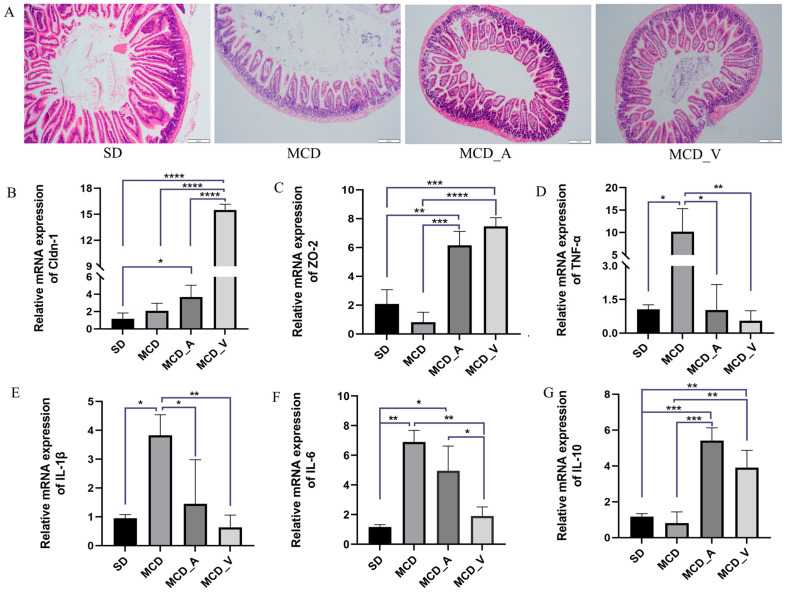
Probiotics improve gut barrier function. (**A**) Morphological analysis of intestine tissues by HE staining; (**B**). The expression of Cldn-1 in livers of mice; (**C**) The expression of ZO-2 in livers of mice; (**D**–**F**) Expression levels of hepatic cytokines TNF-α, IL-1β, and IL-6, as determined by RT-PCR; (**G**) The expression of IL-10 in livers of mice. Values are expressed as mean ± SD, *n* = 10; * *p* < 0.05; ** *p* < 0.01; *** *p* < 0.001; **** *p* < 0.0001.

HE staining was used, together with an analysis of the expression of several tight junction protein genes associated to the gut barrier, to determine how these probiotics affected the gut barrier, as shown in Figure 3A. The SD group had intact intestinal tissue structure under a microscope, free of necrosis and edema. The mice fed with MCD exhibited disrupted villi, injured epithelia, and infiltration of inflammatory cells in mucosa. However, these changes in the MCD group were dramatically reversed by the application of either *A. muciniphila* or VSL#3. To ascertain alterations in the tight junction proteins in the barrier, such as claudin 1 (Cldn-1) and zonula occludens-2 (ZO-2), RT-qPCR was performed. The findings showed that, in comparison to mice in the SD and MCD groups, the mice in MCD_A and MCD_V groups had markedly higher expression levels of Cldn-1 and ZO-2 (Figure 3B,C). Additionally, the expression of Cldn-1 was higher in MCD_V than in the MCD_A group. Furthermore, Figure 3D,E show that there was a considerable rise in the MCD group’s expression of proinflammatory cytokines, including TNF-α and IL-1β, as compared to SD group, in addition to a marked drop in the MCD_A and MCD_V groups. As indicated in Figure 3F, the *A. muciniphila* supplement did not influence the liver IL-6 expression, but the same reduced significantly after VSL#3 intake. There was no significant change in the expression of IL-8 among the groups (Supplementary Figure S1C). In addition, the probiotic treatment groups showed higher expression of IL-10 (Figure 3G).

### 3.3. A. muciniphila and VSL#3 Treatments Both Changed the Makeup of the Intestinal Flora in NASH Mice

**Figure 4:**
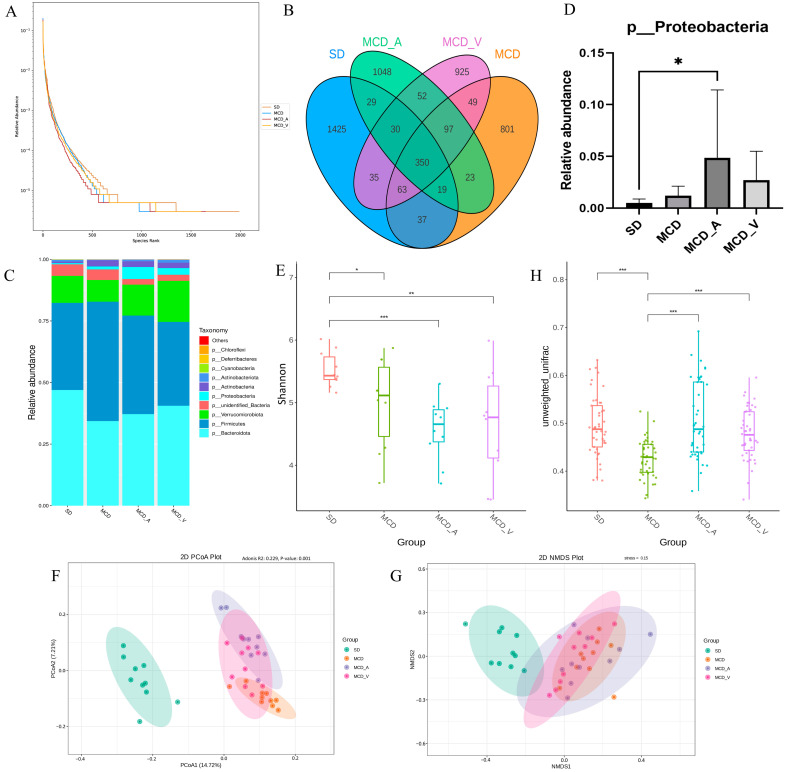
Intestinal microbial diversity and composition among the four groups. (**A**) Rank abundance curves of the four groups; (**B**) Venn diagram of the makeup of ASVs in intestinal flora; (**C**) Average relative abundances of dominant bacterial phyla; (**D**) Relative abundance of Proteobacteria; (**E**) α diversity comparison: Shannon index; (**F**,**G**) β diversity of clustering analysis: PCoA and NMDS analysis of 4 groups at the amplicon sequence variant (ASV) level. (**H**) Statistical analysis based on unweighted UniFrac metrics. PCoA, principal coordinates analysis; NMDS, nonmetric multidimensional scaling analysis. * *p* < 0.05; ** *p* < 0.01; *** *p* < 0.001.

We used 16S rDNA sequencing technology to investigate the variations in the gut flora following an 8-week administration of *A. muciniphila* or VSL#3, given the tight link between gut microbiota and liver disorders mediated by the enterohepatic axis. A total of 3,508,009 raw reads and 3,142,088 effective reads were acquired from the sequencing analysis of fecal microorganisms by filtering and splicing. An identical amplicon sequence variant (ASV) designation was given to any sequences that shared 97% similarity. As shown in Figure 4A, compared with mice fed with an SD, the relative abundance of fecal microbiota was significantly lower in mice fed an MCD, according to the rank abundance curve. A total of 4983 ASVs were found in all fecal samples, as indicated by the Venn diagram, with 350 of those found in every group being classified as core ASVs. While 1425, 801, 1048 and 925 ASVs were uniquely identified within the SD, MCD, MCD_A, and MCD_V groups, respectively, the core ASVs made up around 7% of all ASVs (Figure 4B). We then looked at the phylum-level makeup of the fecal microbiota community (Figure 4C). Based on the taxonomic compositional analysis of the gut microbiota, the phylum-level identification of the main bacterial species in the intestine was made. These species belong to the phyla *Verrucomicrobiota*, *Firmicutes*, *Proteobacteria*, and *Bacteroidota*. In addition, taxonomic bar plots are used in the Supplemental Figure S2A to display the intestinal flora taxonomy for all 40 samples at the phylum level. In Figure 4D, it can be seen that the MCD_A group had a much greater relative abundance of proteobacteria, as compared with the other three groups. Additionally, within each of the four groups, the relative abundances of the *Bacteroidota*, *Firmicutes*, *Verrucomicrobiota*, and *Actinobacteria* phyla were examined (Supplemental Figure S2B–E). As Figure 4E of the α diversity analysis illustrates, the Shannon index, which has a positive correlation with species diversity, significantly dropped in the MCD, MCD_A, and MCD_V groups. Nevertheless, there were no noteworthy distinctions in α diversity between the three groups. This suggested that after feeding on an MCD, the abundance and uniformity of the intestinal flora changed. Microbiota in the SD group and the other three groups were shown to be clustered differently according to β diversity analysis, based on principal coordinate analysis (PCoA) and nonmetric multidimensional scaling (NMDS) analysis. The two primary coordinates (PCoA1 and PCoA2) or nonmetric multidimensional scaling (NMDS1 and NMDS2), as illustrated in Figure 4F,G, indicate the distance between the samples, showing that those positioned close together have more similar compositions. Boxplots of β diversity among groups were used to show whether differences were significant. Both the β diversity in MCD_A and that in MCD_V were higher than in the MCD group; furthermore, the β diversity in MCD_A was higher than in the SD group (Figure 4H). These findings showed that probiotic supplementation might alter the diversity, richness, and abundance of the fecal microbiota.

**Figure 5:**
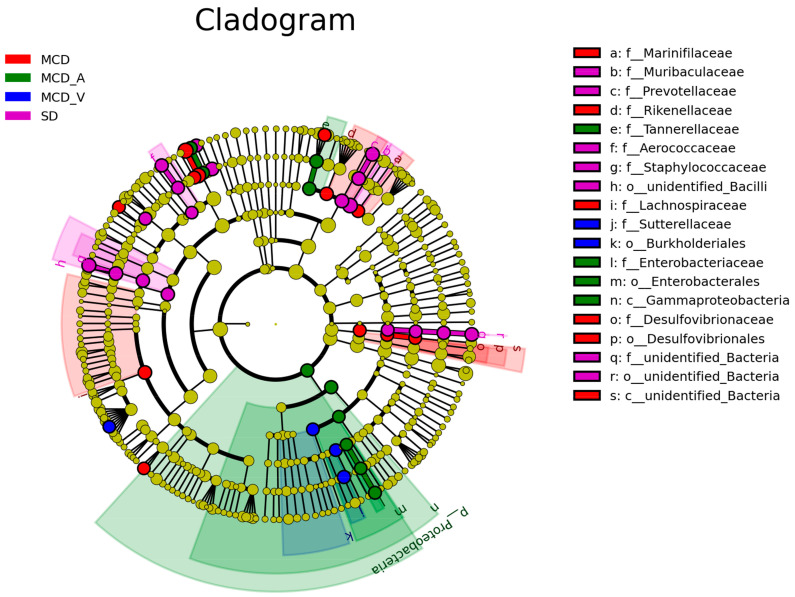
LEfSe analysis among the four groups: Cladogram representation of the significantly different taxa features, from phylum (inner circle) to genus (outer circle). LEfSe: linear discriminant analysis (LDA) effect size analysis.

Next, as demonstrated in Supplemental Figures S3 and S5, a linear discriminant analysis (LDA) effect size (LEfSe) study was conducted to further investigate the variations among the four groups of intestinal microbial communities. The LEfSe approach, which can be used to identify variations in bacterial abundance, combines LDA with a nonparametric test. In the SD group, the most significant genera were *Muribaculaceae*, *Prevotellaceae*, *Aerococcaceae*, and *Staphylococcus saprophyticus*; in the MCD group, the major populations were *Ileibacterium valens*, *Marinifilaceae*, *Turicibacter*, *Rikenellaceae*, and *Desulfovibrionaceae*. Our results also indicated that *Tannerellaceae*, *Enterobacteriaceae*, *Gammaproteobacteria*, and *Faecalibaculum rodentium* dominated the MCD_A group. *Sutterellaceae*, *Parasutterella*, and *Burkholderiales* were dominant in the MCD_V group, which was different from the SD and MCD groups (Figure 5).

### 3.4. Phylogenetic Investigation of Communities by Reconstruction of Unobserved States (PICRUSt) to Predict Microbial Functional Pathways

**Figure 6:**
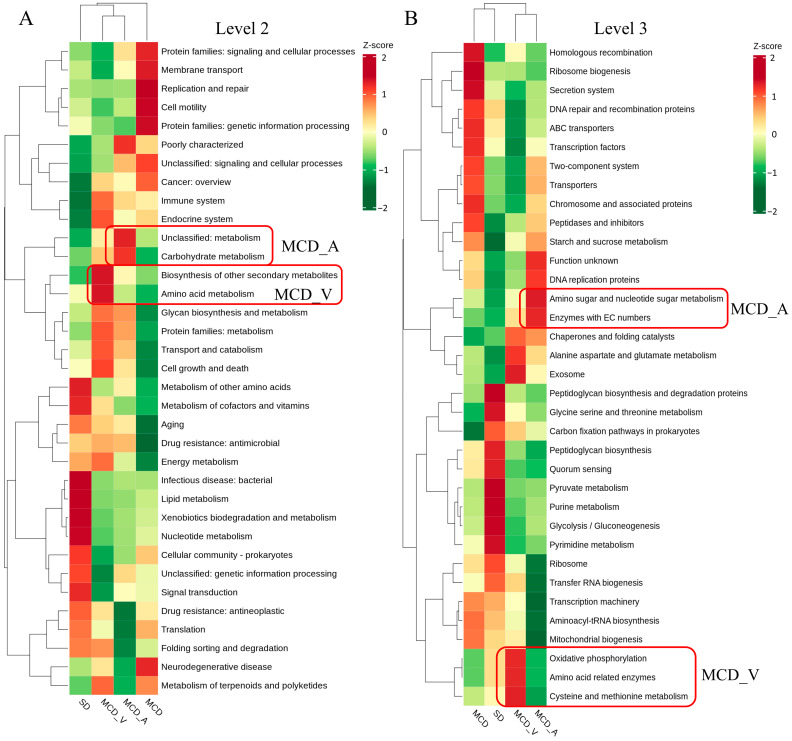
Relative abundance functional comparisons by PICRUSt2 among the four groups, based on the Kyoto Encyclopedia of Genes and Genomes (KEGG) database. (**A**) Level 2 function categories. (**B**) Level 3 function categories.

Prior studies have pointed out a connection between the gut flora and the genesis of NAFLD. In the present study, we used the Kyoto Encyclopedia of Genes and Genomes (KEGG) database for additional analysis subsequent to the PICRUSt2 program to explore the changes in gut flora activities. The functional relative abundance cluster heatmap of the KEGG-Level-2 categories, as displayed in Figure 6A, demonstrated a preferential abundance of carbohydrate metabolism and some other unclassified metabolism in the MCD_A group, and biosynthesis of other secondary metabolites, amino acid metabolism and glycan biosynthesis and metabolism in the MCD_V group. However, replication and repair, cell motility, and protein families in genetic information processing were more observed in the MCD group. For the SD group, bacterial infectious disease, lipid metabolism, xenobiotics biodegradation and metabolism and nucleotide metabolism were more abundant. As illustrated in Figure 6B, based on KEGG-Level-3 prediction, the MCD_A group exhibited a preferential abundance of amino sugar and nucleotide sugar metabolism and enzymes with EC numbers; exosome, cysteine and methionine metabolism, amino acid related enzymes and oxidative phosphorylation were more evident in the MCD_V group. The MCD group still displayed categories related to DNA repair and recombination proteins, as well as ribosome biogenesis, homologous recombination, and the secretion system. Pyruvate metabolism, peptidoglycan biosynthesis and degradation proteins, purine metabolism, glycolysis/gluconeogenesis, and pyrimidine metabolism were enriched in the SD group (Figure 6B). The predicted microbial functional pathways in MCD_A, MCD_V, and SD groups were more enriched in metabolic-related pathways, indicating that probiotics administration restored the normal function of intestinal flora to some extent. Furthermore, carbohydrate metabolism was more important in the MCD_A group; however, amino acid metabolism and oxidative phosphorylation were more important in MCD_V group.

### 3.5. Alterations in Metabolism among the Four Groups Induced by Diets and Probiotics Supplement

**Figure 7:**
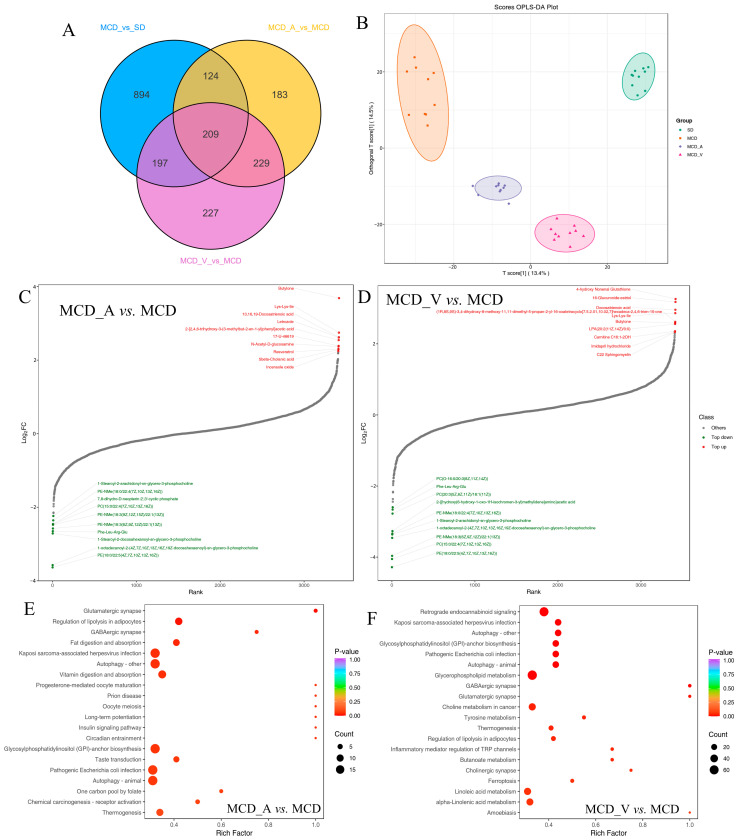
Metabolic characteristics in the four groups. (**A**) Venn diagram of the changed metabolites corresponding to groups MCD_A versus MCD, MCD_V versus MCD, and MCD versus SD; (**B**) OPLS-DA score plots of metabolites. Dynamic distribution of metabolite content differences: (**C**) MCD_A versus MCD and (**D**–**F**) MCD_V versus MCD. Metabolic analysis is based on the KEGG database, and enriched pathways are displayed by bubble plots (MCD_A versus MCD, MCD_V versus MCD).

Liver tissue samples were analyzed by untargeted LC/MS. After quality control, data filtering, and normalization, we identified 3727 metabolic features across 40 samples. The differential metabolites in the corresponding comparison groups MCD_A versus MCD, MCD_V versus MCD, and MCD versus SD are as shown in Figure 7A; 209 altered metabolites were shared among these groups. Variable importance in projection (VIP), fold change (FC), and *p*-value were utilized to choose differential metabolites between different groups in order to evaluate the overall metabolic differences. Supplementary Figure S4A–C shows the screening findings. A total of 1424 metabolites with marked differences were discovered between MCD and SD (850 upregulated and 574 downregulated), MCD_A and MCD (745 upregulated and 343 downregulated), and MCD_V and MCD (863 upregulated and 305 downregulated). As illustrated in Figure 7B, according to PCA and orthogonal partial least squares-discriminant analysis (OPLS-DA), the metabolites in the liver were significantly different according to dietary content and probiotics supplement. Additionally, the metabolites among the three comparison groups were calculated based on the value of fold change, and the top 10 up-regulated and down-regulated metabolites were marked (Figure 7C,D). Compared with the mice in MCD group, glycerophospholipids, including phosphatidyl ethanolamine (PE) and phosphatidylcholine (PC), were outlier down-regulated, and butylone and Lys-Lys-Ile were outlier upregulated metabolites in MCD_A and MCD_V mice, as shown in Figure 7C,D. Devapamil and ursocholic acid were upregulated and Carnitine C7: DC, (±)12-HEPE were outlier down-regulated metabolites in the MCD group (Supplemental Figure S4D). To investigate the potential functions of altered metabolites, we first annotated metabolic features by using the KEGG database (https://www.genome.jp/kegg/, accessed on 30 November 2023). Figure 7E,F summarizes the enriched metabolic pathways within groups fed with MCDs. These findings demonstrated that the *A. muciniphila* gavage had a significant impact on enriched metabolic pathways, including glutamatergic synapse, glutamatergic synapse, gabaergic synapse, fat digestion and absorption, autophagy, glycosylphosphatidylinositol (GPI)−anchor biosynthesis, and pathogenic *Escherichia coli* infection in the liver. The MCD_V group had a high enrichment in retrograde endocannabinoid signaling, autophagy-other, glycosylphosphatidylinositol (GPI)−anchor biosynthesis, glycerophospholipid metabolism, choline metabolism in cancer, and linolenic acid metabolism. Furthermore, as seen in Supplemental Figure S4E, mice given the MCD diet as compared to the SD diet displayed notable changes in important pathways related to primary bile acid biosynthesis, ferroptosis, sphingolipid signaling pathway, alcoholic liver disease, glutathione metabolism, and purine metabolism.

### 3.6. Analysis of the Cross-Correlation between Metabolites and Intestinal Flora

**Figure 8:**
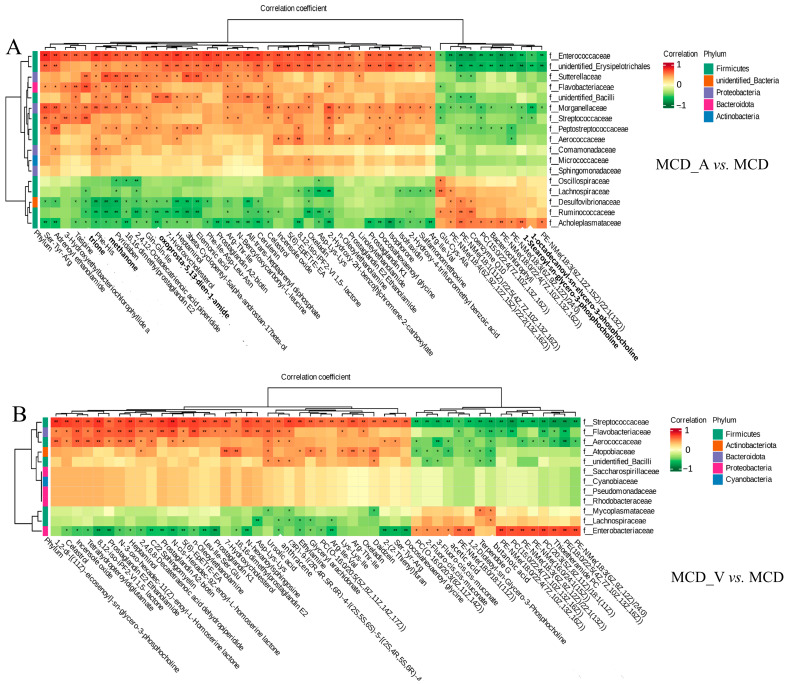
The close correlations between the relative abundance of gut microbiota and altered metabolites. (**A**) The close correlations between the relative abundance of the main family in the gut microbiota and the altered metabolites between the MCD_V and MCD groups; (**B**) The close correlations between the relative abundance of the main family in the gut microbiota and the differential metabolites between the MCD_V and MCD groups. * *p* < 0.05; ** *p* < 0.01.

An examination of correlation was conducted to ascertain the possibility of correlation between the modified microorganisms and metabolites. As indicated in Figure 8A, it was discovered that there are strong relationships between particular bacteria and different metabolites. *Acholeplasmataceae* and *Ruminococcaceae* exhibited a strong positive correlation with glycerophospholipids at the family level, whereas bacteria belonging to the *Enterococcaceae*, unidentified *Erysipelotrichales*, *Morganellaceae*, and *Peptostreptococcaceae* groups showed a negative correlation with these glycerophospholipids between the MCD_A and MCD groups. However, Figure 8B showed that *Enterobacteriaceae* was positively correlated with several kinds of PE and PC between the MCD_V and MCD groups. *Streptococcaceae*, *Flavobacteriaceae*, *Aerococcaceae*, and *Atopobiaceae* were negatively correlated with these glycerophospholipids at the family level. Furthermore, as Supplemental Figure S5 illustrates, there were no noteworthy associations found in glycerophospholipids and gut microbiota between MCD and SD groups at the family level. These findings indicated a close link between intestinal flora and the metabolism of glycerophospholipids. Additionally, in mice given MCD, the supplements of *A. muciniphila* or VSL#3 changed both the gut microbiota and liver metabolism.

## 4. Discussion

Using 16S rDNA gene sequencing and LC-MS/MS techniques, gut microbiota and liver metabolic properties in SD and MCD mice with and without probiotic supplementation were assessed. Both *A. muciniphila* and VSL#3 improved NASH in mice by remodeling the intestinal flora and host metabolism. However, according to our research, *A. muciniphila* had a stronger effect on reducing liver lipid accumulation and VSL #3 performed better in improving the intestinal barrier and reducing inflammation.

Previous research has demonstrated that people with metabolic disorders, such as obesity and metabolic dysfunction-associated fatty liver disease (MAFLD), had lower abundances of *Akkermansia muciniphila* [33]. In obese mice fed with a special diet, supplementation with *A. muciniphila* effectively reversed hepatic steatosis and lowered serum TG [24,27]. Apart from the strain of *A. muciniphila*, there have also been reports of beneficial therapeutic effects on fatty liver caused by Amuc_1100, a particular protein that was extracted from the outer membrane of the bacteria, and extracellular vesicles obtained from *A. muciniphila* [25,26]. In terms of VSL#3, it has been noted that VSL#3 is often used to treat colitis, including ulcerative colitis [29]. In Velayudham’s study, while it was unable to stop liver steatosis and inflammation, it did manage hepatic fibrosis caused by the MCD diet [31]. On the other hand, VSL#3 enhanced insulin sensitivity and decreased the amount of fat in the liver in FXR deletion C57BL/6 mice given a Western diet [32]. In ob/ob mice fed a high-fat diet, Li et al. have shown that the treatment of VSL#3 improved liver histology, lowered hepatic total fatty acid content, and decreased blood ALT levels [34]. The results of the animal study were inconsistent, possibly because they used different animal models and doses of probiotics. Our results demonstrated that oral administration of probiotics could alleviate NASH. In the present study, both *A. muciniphila* and VSL#3 alleviated liver damage, improved liver function (Figure 1D), and reduced hepatic inflammation and lipid accumulation (Figure 2A–F). The common features of NAFLD-related dyslipidemia are hypertriglyceridemia, increased LDL concentration, and decreased HDL concentration, which is consistent with the lipid profile of MCD in the present study [35]. Despite the lack of a significant difference in serum TG between groups MCD_A and MCD_V (Figure 1E), the level of LDL the amount of hepatic fat in the liver was shown to be reduced more by *A. muciniphila* than by VSL#3, using oil red O staining (Figure 2C). Probiotics also improved the function of the intestinal barrier, which may have contributed to their protective effects on the liver (Figure 3A–F). Additionally, VSL #3 has a stronger ability to restore intestinal barrier function than *A. muciniphila*.

The intestinal microbiota has obtained recognition in recent years as one of the principal environmental elements that significantly influences the host’s health. Two prominent phylum members of the gut microbiota are Firmicutes and Bacteroidetes, which are frequently linked to a wide range of illnesses [34,36]. In our investigation, there was no discernible difference in the relative abundance of Firmicutes and Bacteroidetes across the four groups (Supplemental Figure S1). In contrast to earlier research, the treatment of *A. muciniphila* increased the relative abundance of Proteobacteria (Figure 4D). Prior data that have been released indicate that Proteobacteria are more prevalent in NAFLD patients [37,38]. This may have been caused by different sample selections. The variations in intestinal flora between mice in the SD group and mice fed MCD were identified by 16S rDNA gene sequencing. We found that α diversity decreased significantly in the MCD group, regardless of whether probiotics were administered (Figure 4E). However, β diversity analysis indicated that the four groups were well separated (Figure 4F–G). There were notable distinctions between the gut microbiota of NASH mice and healthy control mice when it came to classification and composition. Furthermore, the MCD diet’s reduction in β diversity was recovered by supplements containing *A. muciniphila* or VSL#3 (Figure 4F). These outcomes demonstrated the validity of our system and its applicability to biomarker screening.

The intestinal flora among the four groups is compared using LEfSe analysis, which facilitates the development of biomarkers. *Ileibacterium valens* is a member of the family *Erysipelotrichaceae* and is linked to metabolic health; it has been shown to increase dramatically following the introduction of a high-fat, high-sucrose diet [39]. It has been reported recently that *Ileibacterium valens* promotes intestinal inflammation and tumorigenesis in colorectal cancer [40,41]. Previous research indicated that the abundance of *Marinifilaceae* increased in mice fed with a long-term methionine restriction diet [42]. *Turicibacter* was abundantly detected in tumor-bearing mice [43]. In addition, *Desulfovibrio* and *Turicibacter* were considered pro-inflammatory taxa in David Ma’s study [44]. The role of *Rikenellaceae* in human disease is complex. Bian et al. discovered a negative correlation between *Rikenellaceae* and pro-inflammatory cytokines, as well as other injury factors [45]. The present study showed enrichment of *Ileibacterium valens*, *Marinifilaceae*, *Turicibacter*, and *Desulfovibrionaceae* in the MCD group (Figure 5 and Supplemental Figure S2). Consistent with our findings, it was observed that the combination of probiotics and dietary fiber greatly boosted the abundance of *Tannerellaceae*. In summary, our findings suggest that in mice with NASH, *A. muciniphila* can decrease the quantity of detrimental microbiota produced by MCD and increase the abundance of beneficial bacteria in their guts.

Intestinal flora regulates the growth process and the occurrence of chronic diseases by influencing various metabolic responses of the host [46]. The PICRUst2 algorithm was utilized to further examine the metabolic activities of gut flora. Based on our findings, the microbiota in the MCD group was more obviously enriched in replication and repair, cell motility, neurodegenerative disease, ribosome biogenesis, secretion system, and DNA repair and recombination proteins (Figure 6A,B). However, the microbiota in the level 2 and level 3 groups of the SD, MCD_A, and MCD_V groups was considerably enriched in pathways associated with several metabolic pathways, including, but not limited to, lipid metabolism, xenobiotics biodegradation and metabolism, nucleotide metabolism, amino acid metabolism, carbohydrate metabolism, biosynthesis of other secondary metabolites, and oxidative phosphorylation. The results suggested that the physiological activities and structures of NASH mice induced by MCD were severely damaged; however, oral-gavage *A. muciniphila* or VSL#3 could mitigate damage, restore and maintain normal metabolic activity. Furthermore, the microbiota of MCD_A group was more enriched in carbohydrate metabolism, while the amino acid metabolism and oxidative phosphorylation were more obvious in MCD_V group.

In terms of metabolomics, the present research revealed that compared with healthy controls, NASH mice had unique metabolic characteristics, and the application of probiotics changed their metabolite profiles. Among the four groups, a total of 2063 differently accumulating metabolites were found. The samples from the SD-, MCD-, and probiotics-treated groups were segregated from one another and clustered together within groups (Figure 7B) according to the OPLS-DA analysis, and each group had a distinct metabolic profile. This study found that the levels of glycerophospholipids were noticeably decreased after administration of probiotics, mainly including PC and PE (Figure 7C,D). Glycerophospholipids are major components of cellular membranes [47]. Furthermore, since lipid droplets only contain one phospholipid membrane, the proportion of PC and PE on the droplet surface has a significant impact on how dynamically the lipid droplets vary. It was reported that the PC/PE ratio in the liver was correlated with the severity of NAFLD [48]. The present investigation has suggested that both *A. muciniphila* and VSL#3 affected glycerophospholipid metabolism, although their exact significance in NAFLD remains to be further defined.

The KEGG enrichment analysis showed that the differential metabolites were mainly involved in autophagy, regulation of lipolysis in adipocytes, fat digestion and absorption, glycosylphosphatidylinositol (GPI)−anchor biosynthesis, pathogenic *Escherichia coli* infection, and thermogenesis in the MCD_A group, compared with the MCD group (Figure 7E). The metabolites were more enriched in retrograde endocannabinoid signaling, autophagy, glycerophospholipid metabolism, choline metabolism in cancer, and linoleic acid metabolism in mice gavage with VSL#3 (Figure 7F). Autophagy is a process by which cells degrade themselves and recycle intracellular components; it is involved in the development of NAFLD and controls lipid metabolism [49]. Complex glycolipids known as glycosylphosphatidylinositols (GPIs) serve as membrane anchors for a variety of eukaryotic cell surface proteins and are involved in a number of biological processes, including signal perception, adhesion, material transport, and surface metabolism [50]. The results of functional analysis further indicated that *A. muciniphila* and VSL#3 could restore the metabolism in MCD mice, especially lipid metabolism.

Through Spearman’s correlation analysis (Figure 8), we found that some intestinal floras are closely related to the metabolism of glycerophospholipids. The joint analysis does not, however, allow for the establishment of a firm relationship, and additional study will be required in the future to fully comprehend the mechanisms at play.

There are some limitations to our study. First, additional clinical investigations are required to validate the correlation between the reported differences in intestinal flora and metabolic characteristics in this study, as animal models have limitations. Furthermore, we showed that probiotics control metabolism, particularly glycerophospholipid metabolism, and gut microbiota, but we did not look into the exact mechanism. In that area, more thorough and perceptive research projects need to be planned and carried out.

## 5. Conclusions

In conclusion, our research showed that both *A. muciniphila* and VSL#3, when given to MCD mice, reduced liver lipid deposition and improved intestinal barrier function. However, *A. muciniphila* performed better in decreasing lipid accumulation and VSL#3 had a stronger ability to improve intestinal ecology. Probiotics taken orally not only improved the abundance of the gut flora but also restored the host’s metabolic equilibrium, particularly the regulation of lipid metabolism. Significantly, in clinical practice, these probiotics may be used as a treatment therapy for NASH.

## Figures and Tables

**Table 1:** Primer sequences for the genes detected.

Gene Name	Forward Primer Sequence (5′-3′)	Reverse Primer Sequence (5′-3′)
Cldn-1	GGGGACAACATCGTGACCG	AGGAGTCGAAGACTTTGCACT
ZO-2	ATGGGAGCAGTACACCGTGA	TGACCACCCTGTCATTTTCTTG
IL-1β	GAAATGCCACCTTTTGACAGTG	TGGATGCTCTCATCAGGACAG
TNF-α	CCTGTAGCCCACGTCGTAG	GGGAGTAGACAAGGTACAACCC
IL-6	CTGCAAGAGACTTCCATCCAG	AGTGGTATAGACAGGTCTGTTGG
IL-8	CAAGGCTGGTCCATGCTCC	TGCTATCACTTCCTTTCTGTTGC
IL-10	GCTCTTACTGACTGGCATGAG	CGCAGCTCTAGGAGCATGTG
GAPDH	AGGTCGGTGTGAACGGATTTG	TGTAGACCATGTAGTTGAGGTCA

## Data Availability

The raw data of the sequencing were uploaded to the Sequence Read Archive (SRA) database of NCBI (PRJNA1097335). The raw data for the untargeted metabolomics will be uploaded to the MetaboLights website.

## Supplementary Materials

The following supporting information can be downloaded at: https://www.mdpi.com/article/10.3390/microorganisms12051020/s1, Figure S1: The expression of TNF-α and IL-8 in livers or gut of mice; Figure S2: Relative abundances of dominant bacterial at phylum level; Figure S3: LEfSe analysis: Histogram showing the LDA scores of genera differentially abundant among the four groups; Figure S4: Metabolic characteristics in four groups; Figure S5: The close correlations between the relative abundance of the main family in the gut microbiota and the differential metabolites between the MCD and SD groups.

## References

[1] Cholongitas E., Pavlopoulou I., Papatheodoridi M., Markakis G.E., Bouras E., Haidich A.-B., Papatheodoridis G. (2021). Epidemiology of nonalcoholic fatty liver disease in Europe: A systematic review and meta-analysis. Ann. Gastroenterol..

[2] Rojano A., Sena E., Manzano-Nuñez R., Pericàs J.M., Ciudin A. (2023). NAFLD as the metabolic hallmark of obesity. Intern. Emerg. Med..

[3] Younossi Z., Tacke F., Arrese M., Chander Sharma B., Mostafa I., Bugianesi E., Wai-Sun Wong V., Yilmaz Y., George J., Fan J. (2019). Global perspectives on nonalcoholic fatty liver disease and nonalcoholic steatohepatitis. Hepatology.

[4] Raza S., Rajak S., Anjum B., Sinha R.A. (2019). Molecular links between non-alcoholic fatty liver disease and hepatocellular carcinoma. Hepatoma Res..

[5] Fang J., Yu C.-H., Li X.-J., Yao J.-M., Fang Z.-Y., Yoon S.-H., Yu W.-Y. (2022). Gut dysbiosis in nonalcoholic fatty liver disease: Pathogenesis, diagnosis, and therapeutic implications. Front. Cell. Infect. Microbiol..

[6] Ore A., Akinloye O.A. (2021). Phytotherapy as multi-hit therapy to confront the multiple pathophysiology in non-alcoholic fatty liver disease: A systematic review of experimental interventions. Medicina.

[7] Hoozemans J., de Brauw M., Nieuwdorp M., Gerdes V. (2021). Gut microbiome and metabolites in patients with NAFLD and after bariatric surgery: A comprehensive review. Metabolites.

[8] Nawrot M., Peschard S., Lestavel S., Staels B. (2021). Intestine-liver crosstalk in Type 2 Diabetes and non-alcoholic fatty liver disease. Metabolism.

[9] Jadhav K., Cohen T.S. (2020). Can you trust your gut? Implicating a disrupted intestinal microbiome in the progression of NAFLD/NASH. Front. Endocrinol..

[10] Alisi A., Bedogni G., Baviera G., Giorgio V., Porro E., Paris C., Giammaria P., Reali L., Anania F., Nobili V. (2014). Randomised clinical trial: The beneficial effects of VSL# 3 in obese children with non-alcoholic steatohepatitis. Aliment. Pharmacol. Ther..

[11] Han Y., Ling Q., Wu L., Wang X., Wang Z., Chen J., Zheng Z., Zhou Z., Jia L., Li L. (2023). *Akkermansia muciniphila* inhibits nonalcoholic steatohepatitis by orchestrating TLR2-activated γδT17 cell and macrophage polarization. Gut Microbes.

[12] Naudin C.R., Maner-Smith K., Owens J.A., Wynn G.M., Robinson B.S., Matthews J.D., Reedy A.R., Luo L., Wolfarth A.A., Darby T.M. (2020). Lactococcus lactis subspecies cremoris elicits protection against metabolic changes induced by a western-style diet. Gastroenterology.

[13] Chong C.Y.L., Orr D., Plank L.D., Vatanen T., O’Sullivan J.M., Murphy R. (2020). Randomised double-blind placebo-controlled trial of inulin with metronidazole in non-alcoholic fatty liver disease (NAFLD). Nutrients.

[14] Vrieze A., Van Nood E., Holleman F., Salojärvi J., Kootte R.S., Bartelsman J.F., Dallinga–Thie G.M., Ackermans M.T., Serlie M.J., Oozeer R. (2012). Transfer of intestinal microbiota from lean donors increases insulin sensitivity in individuals with metabolic syndrome. Gastroenterology.

[15] Sanders M.E. (2008). Probiotics: Definition, sources, selection, and uses. Clin. Infect. Dis..

[16] Szajewska H., Hojsak I. (2020). Health benefits of *Lactobacillus rhamnosus* GG and Bifidobacterium animalis subspecies lactis BB-12 in children. Postgrad. Med..

[17] Zhao C., Liu L., Liu Q., Li F., Zhang L., Zhu F., Shao T., Barve S., Chen Y., Li X. (2019). Fibroblast growth factor 21 is required for the therapeutic effects of *Lactobacillus rhamnosus* GG against fructose-induced fatty liver in mice. Mol. Metab..

[18] Mobini R., Tremaroli V., Ståhlman M., Karlsson F., Levin M., Ljungberg M., Sohlin M., Bertéus Forslund H., Perkins R., Bäckhed F. (2017). Metabolic effects of L actobacillus reuteri DSM 17938 in people with type 2 diabetes: A randomized controlled trial. Diabetes Obes. Metab..

[19] Hsu Y.-J., Wu M.-F., Lee M.-C., Huang C.-C. (2021). Exercise training combined with Bifidobacterium longum OLP-01 treatment regulates insulin resistance and physical performance in db/db mice. Food Funct..

[20] Oniszczuk A., Oniszczuk T., Gancarz M., Szymańska J. (2021). Role of gut microbiota, probiotics and prebiotics in the cardiovascular diseases. Molecules.

[21] Sanchez-Rodriguez E., Egea-Zorrilla A., Plaza-Díaz J., Aragón-Vela J., Muñoz-Quezada S., Tercedor-Sánchez L., Abadia-Molina F. (2020). The gut microbiota and its implication in the development of atherosclerosis and related cardiovascular diseases. Nutrients.

[22] Derrien M., Vaughan E.E., Plugge C.M., de Vos W.M. (2004). *Akkermansia muciniphila* gen. nov., sp. nov., a human intestinal mucin-degrading bacterium. Int. J. Syst. Evol. Microbiol..

[23] Yoon H.S., Cho C.H., Yun M.S., Jang S.J., You H.J., Kim J.-h., Han D., Cha K.H., Moon S.H., Lee K. (2021). *Akkermansia muciniphila* secretes a glucagon-like peptide-1-inducing protein that improves glucose homeostasis and ameliorates metabolic disease in mice. Nat. Microbiol..

[24] Rao Y., Kuang Z., Li C., Guo S., Xu Y., Zhao D., Hu Y., Song B., Jiang Z., Ge Z. (2021). Gut *Akkermansia muciniphila* ameliorates metabolic dysfunction-associated fatty liver disease by regulating the metabolism of L-aspartate via gut-liver axis. Gut Microbes.

[25] Plovier H., Everard A., Druart C., Depommier C., Van Hul M., Geurts L., Chilloux J., Ottman N., Duparc T., Lichtenstein L. (2017). A purified membrane protein from *Akkermansia muciniphila* or the pasteurized bacterium improves metabolism in obese and diabetic mice. Nat. Med..

[26] Ashrafian F., Shahriary A., Behrouzi A., Moradi H.R., Keshavarz Azizi Raftar S., Lari A., Hadifar S., Yaghoubfar R., Ahmadi Badi S., Khatami S. (2019). *Akkermansia muciniphila*-derived extracellular vesicles as a mucosal delivery vector for amelioration of obesity in mice. Front. Microbiol..

[27] Kim S., Lee Y., Kim Y., Seo Y., Lee H., Ha J., Lee J., Choi Y., Oh H., Yoon Y. (2020). *Akkermansia muciniphila* prevents fatty liver disease, decreases serum triglycerides, and maintains gut homeostasis. Appl. Environ. Microbiol..

[28] Cani P.D., Depommier C., Derrien M., Everard A., de Vos W.M. (2022). *Akkermansia muciniphila*: Paradigm for next-generation beneficial microorganisms. Nat. Rev. Gastro. Hepatol..

[29] Kumar M., Kissoon-Singh V., Coria A.L., Moreau F., Chadee K. (2017). Probiotic mixture VSL# 3 reduces colonic inflammation and improves intestinal barrier function in Muc2 mucin-deficient mice. Am. J. Physiol.-Gastrointest. Liver Physiol..

[30] Jones R.B., Alderete T.L., Martin A.A., Geary B.A., Hwang D.H., Palmer S.L., Goran M.I. (2018). Probiotic supplementation increases obesity with no detectable effects on liver fat or gut microbiota in obese Hispanic adolescents: A 16-week, randomized, placebo-controlled trial. Pediatr. Obes..

[31] Velayudham A., Dolganiuc A., Ellis M., Petrasek J., Kodys K., Mandrekar P., Szabo G. (2009). VSL# 3 probiotic treatment attenuates fibrosis without changes in steatohepatitis in a diet-induced nonalcoholic steatohepatitis model in mice. Hepatology.

[32] Jena P.K., Sheng L., Li Y., Wan Y.-J.Y. (2020). Probiotics VSL# 3 are effective in reversing non-alcoholic steatohepatitis in a mouse model. Hepatobiliary Surg. Nutr..

[33] Crovesy L., Masterson D., Rosado E.L. (2020). Profile of the gut microbiota of adults with obesity: A systematic review. Eur. J. Clin. Nutr..

[34] Li Z., Yang S., Lin H., Huang J., Watkins P.A., Moser A.B., DeSimone C., Song X.-Y., Diehl A.M. (2003). Probiotics and antibodies to TNF inhibit inflammatory activity and improve nonalcoholic fatty liver disease. Hepatology.

[35] Heeren J., Scheja L. (2021). Metabolic-associated fatty liver disease and lipoprotein metabolism. Mol. Metab..

[36] Lin P., Ding B., Feng C., Yin S., Zhang T., Qi X., Lv H., Guo X., Dong K., Zhu Y. (2017). Prevotella and Klebsiella proportions in fecal microbial communities are potential characteristic parameters for patients with major depressive disorder. J. Affect. Disord..

[37] Hoyles L., Fernández-Real J.-M., Federici M., Serino M., Abbott J., Charpentier J., Heymes C., Luque J.L., Anthony E., Barton R.H. (2018). Molecular phenomics and metagenomics of hepatic steatosis in non-diabetic obese women. Nat. Med..

[38] Caussy C., Hsu C., Lo M.T., Liu A., Bettencourt R., Ajmera V.H., Bassirian S., Hooker J., Sy E., Richards L. (2018). Link between gut-microbiome derived metabolite and shared gene-effects with hepatic steatosis and fibrosis in NAFLD. Hepatology.

[39] Cox L.M., Sohn J., Tyrrell K.L., Citron D.M., Lawson P.A., Patel N.B., Iizumi T., Perez-Perez G.I., Goldstein E.J.C., Blaser M.J. (2017). Description of two novel members of the family Erysipelotrichaceae: Ileibacterium valens gen. nov., sp. nov. and Dubosiella newyorkensis, gen. nov., sp. nov., from the murine intestine, and emendation to the description of Faecalibacterium rodentium. Int. J. Syst. Evol. Microbiol..

[40] Yachida S., Mizutani S., Shiroma H., Shiba S., Nakajima T., Sakamoto T., Watanabe H., Masuda K., Nishimoto Y., Kubo M. (2019). Metagenomic and metabolomic analyses reveal distinct stage-specific phenotypes of the gut microbiota in colorectal cancer. Nat. Med..

[41] Fu T., Huan T., Rahman G., Zhi H., Xu Z., Oh T.G., Guo J., Coulter S., Tripathi A., Martino C. (2023). Paired microbiome and metabolome analyses associate bile acid changes with colorectal cancer progression. Cell Rep..

[42] Nagarajan A., Lasher A.T., Morrow C.D., Sun L.Y. (2024). Long term methionine restriction: Influence on gut microbiome and metabolic characteristics. Aging Cell.

[43] Zackular J.P., Baxter N.T., Iverson K.D., Sadler W.D., Petrosino J.F., Chen G.Y., Schloss P.D. (2013). The gut microbiome modulates colon tumorigenesis. mBio.

[44] Ma D., Wang A.C., Parikh I., Green S.J., Hoffman J.D., Chlipala G., Murphy M.P., Sokola B.S., Bauer B., Hartz A. (2018). Ketogenic diet enhances neurovascular function with altered gut microbiome in young healthy mice. Sci. Rep..

[45] Bian X., Wu W., Yang L., Lv L., Wang Q., Li Y., Ye J., Fang D., Wu J., Jiang X. (2019). Administration of *Akkermansia muciniphila* ameliorates dextran sulfate sodium-induced ulcerative colitis in mice. Front. Microbiol..

[46] Hoffman D.J., Campos-Ponce M., Taddei C.R., Doak C.M. (2017). Microbiome, growth retardation and metabolism: Are they related?. Ann. Hum. Biol..

[47] Chiu D.-C., Baskin J.M. (2022). Imaging and editing the phospholipidome. Acc. Chem. Res..

[48] Li Z., Agellon L.B., Allen T.M., Umeda M., Jewell L., Mason A., Vance D.E. (2006). The ratio of phosphatidylcholine to phosphatidylethanolamine influences membrane integrity and steatohepatitis. Cell Metab..

[49] Qian H., Chao X., Williams J., Fulte S., Li T., Yang L., Ding W.-X. (2021). Autophagy in liver diseases: A review. Mol. Asp. Med..

[50] Ferguson M.A., Homans S.W., Dwek R.A., Rademacher T.W. (1988). Glycosyl-phosphatidylinositol moiety that anchors Trypanosoma brucei variant surface glycoprotein to the membrane. Science.

