# Supplementary material for: Role of Probiotics in Gut Microbiome and Metabolome in Non-Alcoholic Fatty Liver Disease Mouse Model: A Comparative Study
Source: Microorganisms. 2024 May 17;12(5):1020. doi: 10.3390/microorganisms12051020 (PMC11124503; doi:10.3390/microorganisms12051020)
Supplement: Supplementary file 1 [file microorganisms-12-01020-s001.zip › microorganisms-2984945-supplementary.pdf]

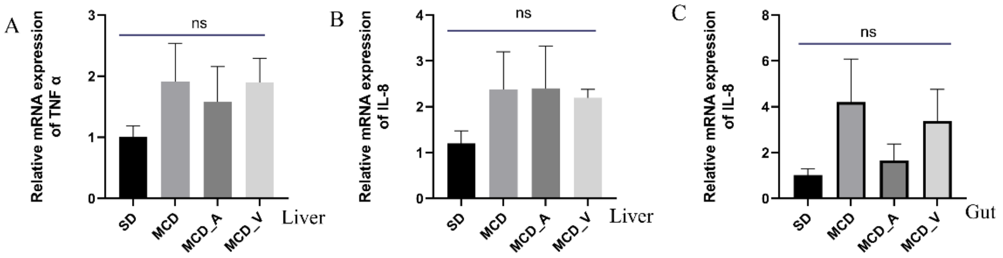

Supplemental Figure S1. The expression of TNF-αand IL-8 in livers or gut of mice. The above values are expressed as mean ± SD, n = 10; ns, no significant difference; *p < 0.05; **p < 0.01; ***p < 0.01; ****p < 0.001.

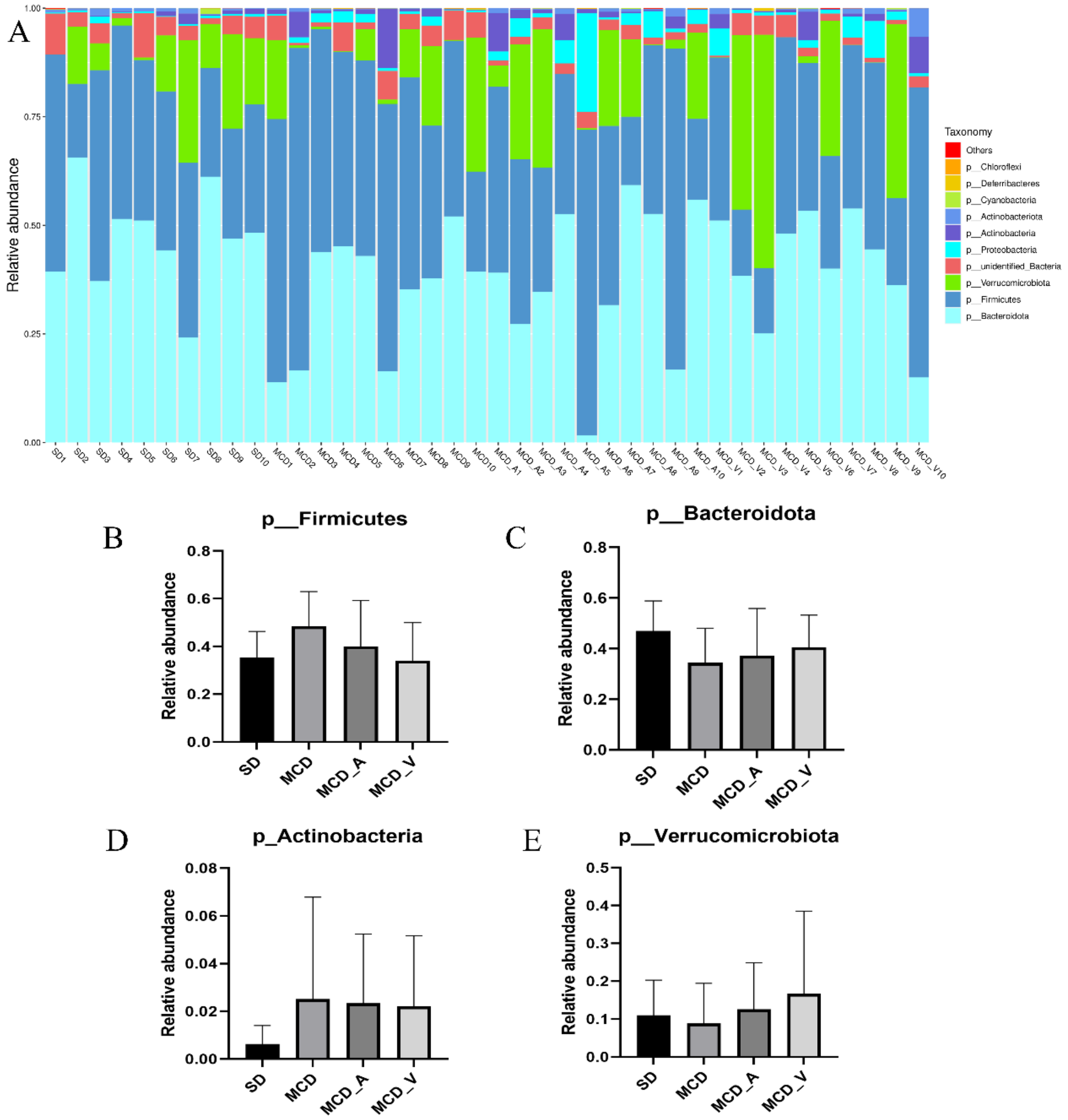

Supplemental Figure S2. Relative abundances of dominant bacterial at phylum level. (A). Individual relative abundances of dominant bacterial phylum level; (B)-(E): Relative abundance of Firmicutes, Bacteroidetes, Actinobacteria, and Verrucomicrobiota.

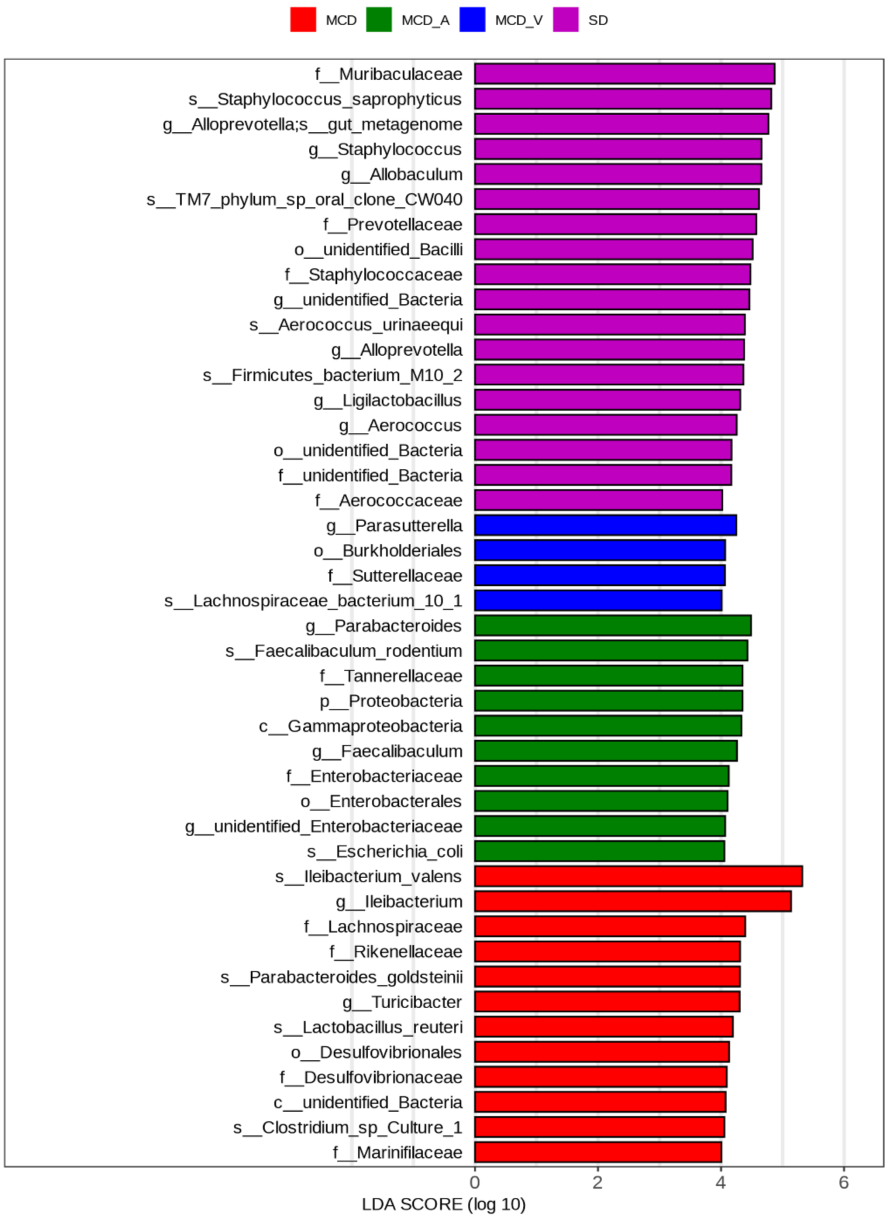

Supplemental Figure S3. LEfSe analysis: Histogram showing the LDA scores of genera differentially abundant among the four groups.

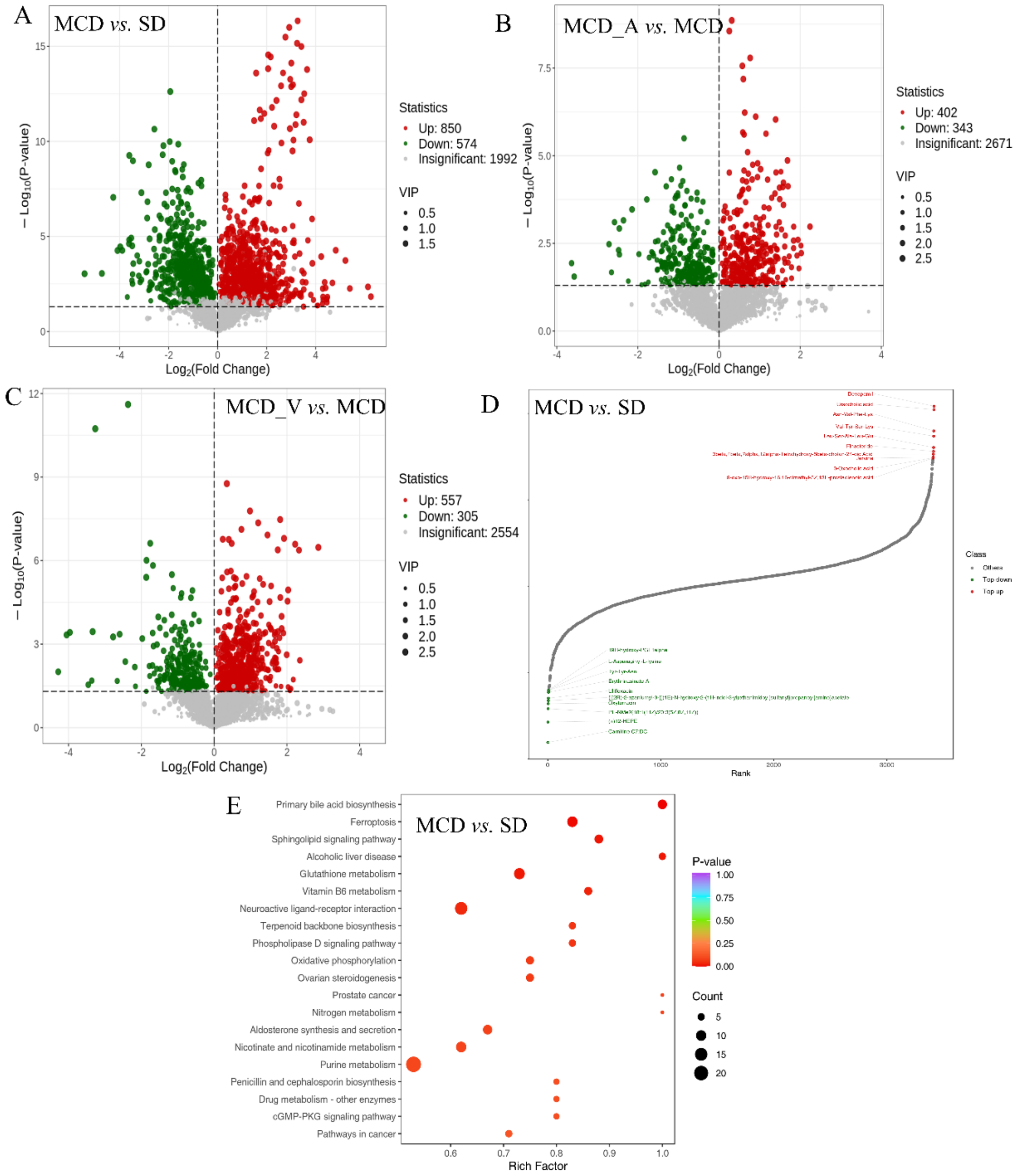

Supplemental Figure S4. Metabolic characteristics in four groups. (A)-(C): Volcano plots showing the differential metabolite levels corresponding to the comparison groups, including MCD versus SD, MCD_A versus MCD, and MCD_V versus MCD. (D). Dynamic distribution of metabolite content differences (MCD versus SD); (E). Metabolic analysis based on the KEGG database and enriched pathways were displayed by bubble plots (MCD versus SD).

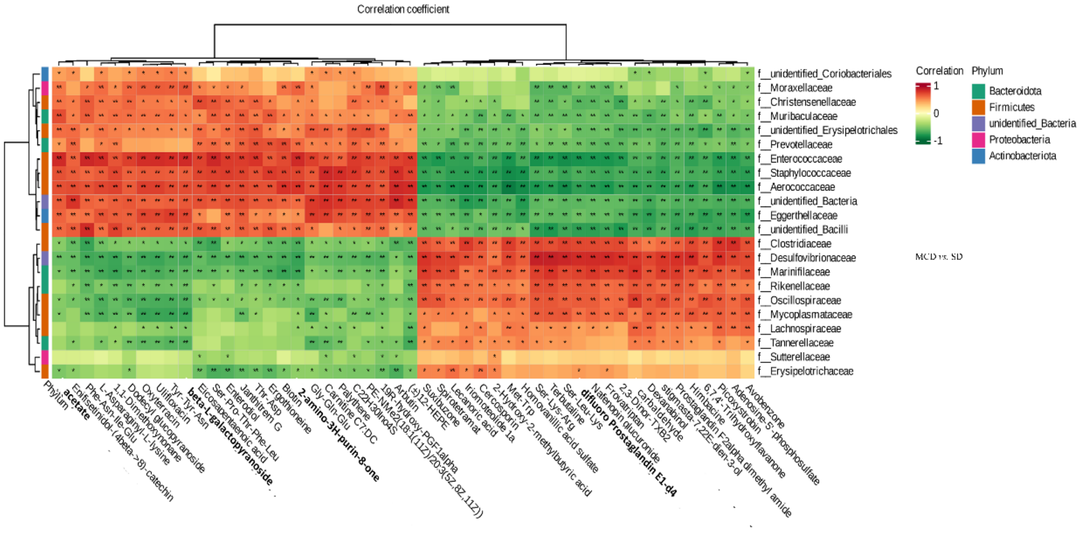

Supplemental Figure S5. The close correlations between the relative abundance of the main family in the gut microbiota and the differential metabolites between the MCD and SD groups
